# Does the Delivery System Matter? The Scaling-Out of a School-Based Resilience Curriculum to the Social Services Sector

**DOI:** 10.3389/fpsyt.2021.578048

**Published:** 2021-05-04

**Authors:** Josefine L. Lilja, Birgitta Kimber, Charli Eriksson, Barbro Henriksson, Therése Skoog

**Affiliations:** ^1^Department of Psychology, University of Gothenburg, Gothenburg, Sweden; ^2^R&D Primary Health Care, Västra Götaland, Sweden; ^3^Department of Clinical Sciences, Umeå University, Umeå, Sweden; ^4^Department of Public Health Sciences, Stockholm University, Stockholm, Sweden; ^5^Junis – Movendi's Junior Association, Stockholm, Sweden

**Keywords:** implementation, resilience curriculum, scaling-out, social services, school

## Abstract

**Background:** The context is highly relevant to the implementation of new health-related programs and is an implicit or explicit part of the major implementation models in the literature. The Resilience Curriculum (RESCUR) program was developed to foster the psychosocial development of children in early and primary education. RESCUR seeks specifically to decrease children's vulnerability. It aims to promote the emotional and social learning of children who may be at risk of leaving school pre-maturely, social exclusion and mental-health problems. The program is taught using a teachers' manual to support consistency of delivery, a parents' guide, and a resource package. This study aimed to examine the scaling-out of RESCUR to social services, and specifically to test if implementation differs between the school and social services sectors.

**Methods:** RESCUR was implemented in schools and social services in Sweden 2017–2019. Data were collected *via* group leaders' self-reports and observation protocols for 3 months after implementation started. There were 34 self-reports from schools, and 12 from the social services sector; 30 observation protocols were collected from schools, and 10 from social services. We examined whether there were differences in implementation outcomes (in, for example, dosage, duration, fidelity, adaptation, quality of delivery) between the two delivery systems. Descriptive statistics were prepared and non-parametric tests of significance conducted to compare implementation-related factors across the two settings.

**Results:** Analyses of both the observation protocols and group leaders' self-reports revealed that RESCUR was well-implemented in both schools and social services. The results showed a few significant differences in the outcomes of implementation between the sectors. First, regarding observations, school staff more often adapted the pace of RESCUR lessons to ensure that the children could understand than did social services staff (p < 0.01). Second, social services staff demonstrated greater interest in students and sensitivity to the needs of individual students than did school staff (p = 0.02). Regarding self-reports, social services staff reported having delivered more (p = 0.4) and longer (p < 0.01) lessons than did school staff. Second, school staff reported greater fidelity to (p = 0.02) and less adaptation of (p < 0.01) the intervention than did social services staff. Both observations and self-reports, however, indicated a high fidelity of implementation.

**Conclusions:** Overall, the findings suggest that the resilience program, designed for delivery in schools, can be scaled-out to social services with its implementation outcomes retained. Further research is needed to test the effectiveness of the program regarding child health-related outcomes.

**Clinical Trial Registration:** National Institute of Health, ClinicalTrials.gov, identifier: NCT03655418. Registered August 31, 2018.

## Introduction

In a social and emotional learning context, implementation has been defined “as putting an innovation into practice in such a way that it meets the necessary standards to achieve the innovation's desired outcomes” [(1), p. 465]. Consequently, it is of central importance to achieve desirable implementation outcomes. Programs that monitor implementation are more effective, with effect sizes up to three times larger than those that do not monitor (2). Furthermore, observational data on implementation are more strongly linked to outcomes than self-reported data (2). Durlak and DuPre state that “because observational data are more objective, it seems preferable to use such information for implementation analyses” [(2), p. 331]. School-based social and emotional learning programs have been found effective in targeting broad protective factors to improve psychosocial development in universal populations (3, 4). Consequently, it seems important to use such programs in social services settings and examine how implementation outcomes in this context differs from those in schools. Using a multi-informant method, the present study aimed to examine the scaling-out of the implementation of a resilience curriculum (RESCUR), and specifically to test whether implementation outcomes differ between the school and social services sectors. The curriculum was designed to foster the psychosocial development of children and was developed for children to overcome disadvantages and obstacles in their psychosocial environment, i.e., to promote their resilience (5, 6). Therefore, it seemed relevant to examine whether RESCUR can be scaled-out to the social services sector, where these children might make up targeted groups and may benefit from receiving the content in a smaller group setting.

Implementation can be described as consisting of eight main characteristics (2): (1) Fidelity is the extent to which an innovation corresponds to the originally intended program (e.g., % of program content delivered); (2) Dosage refers to how much of the original program has been delivered (how many lessons or periods, and time per lesson); (3) Quality means how well different program components have been conducted (e.g., “Are the main features of the intervention delivered clearly and correctly?”); (4) Participant responsiveness refers to the degree to which an intervention maintains the interest of participants; (5) Program differentiation involves the extent to which a program's theory and practices can be differentiated from other programs; (6) Monitoring of comparison conditions involves describing the amount and nature of alternative services received by the participants; (7) Program reach refers to the representativeness of program participants. Finally, (8) Adaptation refers to changes made to the original program during implementation (2). In this study, we chose to examine *Fidelity, Adaptation, Dosage, Quality* and *Participant responsiveness* in the implementation of a didactic and interactive resilience-promoting program for children in two different delivery systems—schools and social services—to gain knowledge about whether the program can be implemented similarly across settings. Knowledge of implementation outcomes across settings may act as a guide to further investment. We chose to focus on these outcomes as they are among the most commonly assessed and discussed aspects of implementation in the literature on preventive interventions aimed at children and adolescents (7–9).

The context is highly relevant to the implementation of new health-related programs, such as RESCUR, and is therefore implicitly or explicitly part of the major implementation models in the literature (1, 2). Meyers et al., when reviewing 25 implementation frameworks, found four phases that constitute what is called the “Quality Implementation Framework (QIF) (1). These steps were: Initial consideration Regarding the Host Setting, Creating a Structure for Implementation, Ongoing Structure Once Implementation Begins, and Improving Future Applications. Nilsen and Bernhardsson (10) recently performed a scoping review that examined frameworks describing the contextual factors involved in implementation outcomes. They found that the most common context dimensions were organizational support (included in all 17 frameworks), financial resources (in 16 frameworks), social relations and support (in 15 frameworks), leadership (in 14 frameworks), and organizational culture and organizational readiness to change (in 12 frameworks). Patients/Participants as a contextual determinant were addressed in 11 of the frameworks. Moreover, the authors found that the frameworks included two types of contextual dimensions: those that function as necessary conditions for implementation, and those that act as driving forces for the achievement of implementation. For example, having resources and time may be favorable conditions for implementation, but they generally need to be combined with organizational support and leadership if implementation is to succeed. Taking these findings into account, it is clear that context is a multi-dimensional concept that needs to be addressed when implementing an intervention in a novel context.

Scaling-out refers to expanding the implementation of an intervention or program to a new population or through a new delivery system (11). The intervention examined in this study, the RESilience CURriculum (RESCUR), also known as Surfing the Waves (12), was created as a universal intervention for implementation in schools by teachers; here, however, we wanted to examine, for the first time, whether it could be scaled out for another delivery system, namely social services. Before scaling-out, there must be sufficient justification for it; that is, any new intervention should be expected to provide benefits similar to those found in earlier trials. Since RESCUR aims to decrease children's vulnerability and promote the emotional and social learning of children who may be at risk of early social exclusion, it seemed relevant to examine whether RESCUR could be scaled-out to the social services sector.

In Sweden, the National Schools Curriculum states that compulsory school: “… should promote understanding of other people and the ability to empathize. Concern for the well-being and development of the individual should permeate all school activity. No one should be subjected to discrimination on the grounds of gender, ethnic affiliation, religion, transgender identity or its expression, sexual orientation, age or functional impairment. All such tendencies should be actively combated” (13). The Swedish Social Services Act (2001) states that social services must work to ensure that children and young people grow up under safe conditions. In their work with children who are suffering, they are obliged to cooperate with, among others, health care, the school, and the police. Social services are the authority in Sweden that conducts child protection investigations and has contact with children and families. For example, many municipalities' social services run support groups for children to meet their rights to information, advice and support when there are problems in the family, such as mental illness and addiction problems (14).

Over the last 20 years, several prevention and/or promotion programs have been used, not only in the USA but also in Europe, to promote resilience in children and youth. These include PATHS (Promoting Alternative Thinking Strategies) from the USA, SEAL (Social and Emotional Aspects of Learning) from the UK, and SET (Social and Emotional Training) from Sweden (15). Outcomes include better mental health and enhanced resilience among children (15–18).

Several of these programs have been examined with regard to implementation. For example, an effectiveness trial of PATHS in a high-risk American urban community suggested that support from school principals and a high degree of classroom implementation contributed to the success of the intervention (19). Also, a study has been performed to assess the relationship between implementation and intervention outcomes (20). How training and implementation led to teacher change was shown regarding SET (21), and that implementation really matters was shown in England, where SEAL was implemented nationally in almost all compulsory schools before 2010 (4). In secondary schools, Humphrey et al. (22) found that there were both barriers and facilitators in implementing SEAL. Staff's “will and skill,” plus the availability of time and resources were found to be important factors in driving implementation forward. It was shown that the quality of implementation was of significant importance to SEAL's effectiveness (4, 22).

Like PATHS and SET but unlike SEAL, RESCUR is a “structured program” in that it is manual-based and requires group leaders to follow a curriculum. It was developed as a cooperative project between researchers from six different universities in Europe. Also like the other programs, RESCUR aims to develop children's resilience by fostering the psychosocial development of children in early and primary education, but it was also specifically designed to meet needs in the current social and economic situation in Europe (5, 6, 12).

RESCUR was funded by the European Commission, and built on evidence (5, 6, 23, 24) about risk and protective factors, and social and emotional learning and resilience. The program is taught using a teachers' manual to support consistency of delivery (3 manuals for teachers—for early years, early primary, and primary school children), a parents' guide and a resource package including, for example, music and mindfulness exercises at www.rescur.eu or www.rescur.se.

Resilience can be described in terms of “positive or protective processes that reduce maladaptive outcomes under conditions of risk” [(17), p. 3]. The concept of resilience refers to the ability to cope with stressors, crises and changes without developing severe mental health issues or lashing out at society (18, 25). Most children who are exposed to stressful environments will develop positively despite the odds against them (26, 27). Research has shown that a key factor is resilience, where the interaction between risk and protective factors results in a variety of development patterns (23, 27, 28). Greenberg divides resilience into three broad categories: “characteristics of the individual, the quality of the child's relationships and broader ecological factors such as quality schools, safe neighborhoods and regulatory services” [(17), p. 3]. More abstractly, the approach involves shifting from a pathogenic or deficit model to a more optimistic and salutogenic way of thinking about strengths and adaptive functioning, which gives a new incentive for the development of preventive and therapeutic interventions (23).

Many studies have shown the effectiveness of resilience interventions (17, 18), and that implementation is particularly important for achieving favorable outcomes (2). When an intervention is implemented, with fidelity, in a setting that is similar to one where it has previously been found to be effective, it is reasonable to anticipate similar beneficial outcomes. Most RESCUR interventions are conducted in schools (12, 29, 30), but we wanted to examine the implementation of the program in an additional setting. The primary aim of the study was to determine if the program could be implemented similarly across settings. Moreover, the study aimed to examine the scaling-out of RESCUR, and, more specifically, to test whether implementation differs between the school and social services sectors.

## Materials and Methods

### Project Description

The current study used data collected between 2017 and 2019 from a comprehensive study of the Resilience Curriculum (RESCUR) in Sweden (12). The study was performed by an NGO (Junis, formerly part of IOGT-NTO's Junior Association, now of Movendi International) in collaboration with researchers at Gothenburg, Umeå and Stockholm universities, and was funded by the Public Health Agency of Sweden (12). The project was implemented in collaboration between practitioners and researchers.

### Design

The study was part of a larger cluster-randomized trial in which the effects of RESCUR were measured over a longer period (12). For this study, RESCUR was implemented in two different sectors. This study uses implementation data, all related to RESCUR staff, collected ~3 months after the start of implementation. A multi-method design was used, which consisted of observational data on the leaders and self-reports by the leaders. The aims were to examine the scaling-out of RESCUR and to compare the quality of its implementation in Swedish schools and within social services.

### Procedure

The schools were recruited at school meetings, meetings of principals, and various conferences, some local and some national. Both the schools and the social services were situated in urban, suburban and rural municipalities. They were spread all over Sweden from the north to the south. The schools had both high and low SES. The sizes of the schools and social services units varied. In some schools all pupils had Swedish as their mother tongue, whereas others had up to 92% of pupils speaking a language other than Swedish. The school groups were not matched with the social service groups. The leaders in the schools ranged from 1 to 6, mainly due to the size of the school. In most classes, the class teacher did the RESCUR lessons, and there was just that one teacher per class. In the social services, there were 2–4 leaders in each office, and there were two leaders per group.

The recruitment of group leaders started more than a year before the project was due to begin. Recruitment was made at school meetings and conferences, through contacts with principals, and at an annual, national meeting of all Sweden's political parties. All the staff involved in the two delivery systems were given 3 days of training in RESCUR. They were trained on the same site in Stockholm: 2 days consecutively and another day after 6 months; teachers and social workers at different times. The leaders were trained by one of the program authors (BK) together with the 4th author of this paper (BH). The program was implemented by professional teachers and social workers, all of whom are referred to here as leaders. The leaders were given supervision once each semester, and also on demand. The leaders were observed and filled out self-reports after ~3 months of implementation. The authors of the manuscript compared the leaders with regard to their scores on the observation forms and their own self-reports. There were two independent observers (the second and fourth authors of this paper, BK and BH); they performed several observations together before they started on the specific observations for the study in order to establish inter-rater reliability. They also discussed different items on the implementation forms in order to make sure that they had the same interpretation of what each item meant. The implementation data were collected from active RESCUR groups from the fall of 2017 to the fall of 2019, after all the groups had worked with the RESCUR theme “Developing Communication Skills.” The study was approved by regional ethics committees in Uppsala and Gothenburg.

### Participants

Fifty-one leaders in 22 schools and 17 leaders in 7 social services units were trained to deliver RESCUR. One school with two leaders was excluded from the study because the leaders did not complete the training course. The program was introduced by 68 leaders into 31 school classes in 16 schools, and into 6 groups in the social services sector in 6 municipalities. All teachers had more than 5 years of teaching experience, some more than 20 years. All but three leaders in the social services were very experienced with more than 5 years of experience in social work. There were 30 complete observations of RESCUR in schools, and 10 in the social services sector. Further, there were 34 completed self-reports on RESCUR implementation in schools, and 12 in the social services sector. The numbers of trained leaders, planned numbers of intervention classes/groups, and actual numbers of intervention classes/groups are presented in Table 1. Schools delivered RESCUR to their own students, while social services recruited children from their own locations (primarily children in difficult situations, e.g., with substance-abusing parents) specifically for their RESCUR groups.

**Table 1:** Number of trained staff (leaders) in RESCUR, planned number of classes, and actual intervention classes.

**Organization**	**Number of trained staff (leaders)**	**Planned number of intervention classes/groups**	**Actual number of intervention classes/groups**
Schools	51	46	31
Social services units	17	7	6

### Intervention

The number of children in the classrooms varied between 15 and 25, depending on the age of the pupils and where the school was located (with fewer children in the classes in rural areas). Groups in social services consisted of 6 or 7 children; in half of the groups, roughly half of the children stopped coming to the sessions, whereas, in the other half, all children continued to come. In both the schools and social services, the leaders used age-appropriate manuals, and they made hardly any adaptations. There was one exception, in that one group in social services changed to a manual for younger children.

The training of the leaders consisted of 3 days of formal training, plus regular observations, feedback, and supervision.

Content of the training

First and second day:

- The theory and the background of the RESCUR program
- Definition of the concept of resilience
- Risk and protective factors
- Content of the program, including the teachers'/leaders' guide and the parents' guide
- How to be a resilient teacher/leader
- Mindfulness theory and practice
- The importance of implementation and factors that enhance implementation
- Introduction to classroom management/ leadership
- The RESCUR study
- Activities from the leaders' manuals prepared and performed by the participants.

Third day:

- Classroom management and leadership
- The parents' guide (they read and discussed the parents' guide in order to find ways to involve the parents)
- Communication methodology with a focus on parents.

During the observations, the observers checked for fidelity dosage/duration, quality of delivery and participant responsiveness, teachers' interpersonal style and skills, modeling and generalization, general teaching style and behavior, and global rating of performance.

### Measures

#### Observation Protocols

All leaders were observed after ~3 months of implementation of RESCUR. We used an adapted Swedish translation of the Promoting Alternative Thinking Strategies (PATHS) observation-of-teachers form (17, 20). The PATHS curriculum is a universal school-based prevention curriculum aimed at reducing behavior problems by promoting the development of social-emotional competence in children during the pre-school and elementary school years. The observation-of-teachers form measures quality and other aspects of implementation. The second author (BK) performed the observations in the schools, and the fourth author (BH) the observations in the social services sector. The observer who observed the teachers is one of the authors of the program (BK). She has a PhD and is also a licensed psychotherapist and teacher and licensed special-ed teacher. The other observer, who observed in social services, is a registered occupational therapist. Interrater reliability was established. Three items in the protocol regarded as not relevant to this study of RESCUR were excluded from the analysis.

The items that were included in the study were: Pacing of RESCUR lessons, Affect and Energy during RESCUR lessons, Openness to consultation, Leader's level of punitive discipline, Leader's interpersonal style, Leader's classroom/group management, Teaching of RESCUR concepts, Modeling and generalization of RESCUR concepts, and Global rating of RESCUR implementation quality. Table 2 contains detailed information about each of the measures collected *via* the observation protocols. Note that a leader-led group activity of any kind counted as a lesson.

**Table 2:** Observation protocol—implementation in schools and social services units after ~3 months.

				**Schools *N* = 30**	**Social services units *N* = 10**			
**Observation outcomes**	**Descriptions of leader-related outcomes**	**Scale anchors**	**Scale range**	***M* (SD)**	***M* (SD)**	***U***	***P***	***Z***
Pacing of lessons	Leaders' adjustment during lessons regarding the pace of instruction based on the students' needs/understanding/attention span	1 = 90% 2 = 75% 3 = 60% 4 <60%	1–4 Reverse scored	3.33 (0.92)	1.20 (0.63)	18.00	<0.01	−4.37
Affect and energy during lessons	Leaders' demonstration over time of genuine and appropriate affect and energy	1 = 90% 2 = 75% 3 = 60% 4 <60%	1–4 Reverse scored	3.60 (0.62)	3.50 (0.71)	139.00	0.68	−0.41
Openness to consultation^a^	5 aspects: Leaders' openness to consultation, meeting the observer, sharing challenges, asking questions, and openness to feedback	Yes/No for each aspect	0–5	3.57 (1.01)	3.90 (0.74)	124.00	0.38	−0.87
Leaders' level of punitive discipline	Leaders' level of punitive discipline	1 = does not use, 4 = uses a lot	1–4	1.07 (0.25)	1.00 (0.00)	140.00	0.41	−0.83
Leaders' interpersonal style^a^	6 aspects: Leaders' level of warmth, physical and mental availability, positive behavior, interest in students, sensitivity toward students' individual needs, individual relationships with students	Yes/No for each aspect	0–6	4.83 (1.29)	5.60 (0.97)	83.00	0.02	−2.25
Leaders' classroom/group management^a^	5 aspects: Leaders' level re. clarity of structure, consistency in discipline, clarity of expectations, effective use of preventive techniques, group and individual reinforcement	Yes/No for each aspect	0–5	2.93 (1.46)	2.90 (1.45)	148.00	0.95	−0.06
Teaching of RESCUR concepts^a^	6 aspects: Leaders' ability to clearly state the purpose of a lesson, relate the lesson to other learning, be well-prepared, understand the concepts being taught, assess student understanding throughout the lesson, have lesson content appropriate to the lesson topic	Yes/No for each aspect	0–6	4.73 (1.44)	4.90 (1.79)	123.50	0.38	−0.88
Modeling and generalization^a^	3 aspects: Leader relates his/her experiences to the students' experiences, leader talks about his/her emotions, leader recognizes the emotions of the students	Yes/No for each aspect	0–3	2.34 (1.40)	2.10 (0.88)	128.00	0.58	−0.56
Global rating of implementation quality	Observers' overall rating of RESCUR implementation quality	1 = poor responsiveness to the intended curriculum, 7 = exemplary delivery of the curriculum	1–7	5.60 (1.16)	5.30 (1.25)	130.50	0.53	−0.63

a *For these outcomes, there were several items that could be answered “Yes” or “No.” The total number of endorsed items for each outcome was calculated and used in the analysis*.

#### Self-Reports

Self-reports by the leaders measured fidelity and the extent to which the intervention was implemented according to Humphrey's guidelines for Headstart in the UK (31). For example, there were items regarding whether any adaptation had been made to the program and about dosage. Quality in delivering RESCUR was analyzed by examining the teaching of RESCUR concepts (31). The two initial questions (concerning adaptation and fidelity) required the leaders to evaluate their overall delivery of the intervention. The items that were included in the study were divided into structural aspects, responsiveness aspects, and teaching aspects. The structural aspects were: Adaptation, Fidelity to guidelines, Adaptation of intervention, Dosage—total lessons, Dosage—lessons interval, and Dosage—time per lesson. The responsiveness aspects were: Children's responsiveness—enthusiasm, Children's responsiveness—engagement, Leader's responsiveness—interest, and Leader's responsiveness—enthusiasm. The teaching aspects were: Teaching RESCUR concepts—Overall goal, Teaching RESCUR concepts—Preparedness, Teaching RESCUR concepts—Purpose, and Teaching RESCUR concepts—Responsiveness (see Table 3).

**Table 3:** Leaders' self-reports—implementation in schools and social services units after 3 months.

				**Schools *N* = 34**	**Social services sector *N* = 12**			
**Self-report outcomes**	**Descriptions of leaders' grading of outcomes**	**Scale anchors**	**Scale range**	***M* (SD)**	***M* (SD)**	***U***	***p***	***Z***
**Structural aspects**								
Adaptation	The overall extent to which the leaders adapted the intervention to their group	1 = not at all, 3 = completely	1–3	1.85 (0.44)	2.08 (0.29)	160.00	0.10	−1.67
Fidelity to guidelines	The overall extent to which the leaders followed the sequence and structure of the intervention as outlined in the guidelines	1 = not at all, 5 = completely	1–5	4.09 (0.67)	3.55 (0.52)	108.00	0.02	−2.34
Adaptation of intervention	The leaders were asked about the extent to which they had made changes to the intervention	1 = never, 5 = always)	1–5	2.15 (0.86)	2.92 (0.29)	96.00	<0.01	−2.94
Dosage—total lessons	The leaders noted the total number of lessons/activities that had been delivered as part of the intervention		Total amount of lessons	6.85 (2.45)	8.42 (1.73)	120.00	0.04	−2.04
Dosage—lessons interval	The leaders noted how often the intervention was implemented	1 = daily, 4 = monthly.	1–4	2.93 (0.25)	3.00 (0.00)	168.00	0.37	−0.91
			Reverse scores					
Dosage—time per lesson	The leaders noted the duration of a typical lesson/activity in minutes		Minutes	46.03 (10.57)	90.00 (0.00)	0.00	<0.01	−5.19
**Responsiveness**								
Children's responsiveness—enthusiasm	How enthusiastic the children had been throughout RESCUR	1 = not at all, 5 = completely	1–5	3.74 (0.57)	3.42 (0.51)	146.00	0.10	−1.67
Children's responsiveness—engagement	How engaged the children had been throughout RESCUR	1 = not at all, 5 = completely	1–5	3.79 (0.52)	3.42 (0.51)	133.00	0.04	−2.03
Leaders' responsiveness —interest	How interesting did the leaders find RESCUR	1 = not at all, 5 = completely	1–5	4.13 (0.62)	3.92 (0.51)	168.50	0.30	−1.03
Leaders' responsiveness—enthusiasm	How enthusiastic had the leaders been throughout RESCUR	1 = not at all, 5 = completely	1–5	3.88 (0.55)	3.83 (0.39)	197.00	0.83	−0.22
**Teaching**								
Teaching RESCUR concepts—overall goal	To what extent did the leaders feel they had knowledge of the goal of the RESCUR program	1 = not at all, 5 = completely	1–5	4.13 (0.62)	4.08 (0.67)	197.50	0.85	−0.18
Teaching RESCUR concepts—preparedness	How well-prepared did the leaders feel when delivering RESCUR	1 = not at all, 5 = completely	1–5	3.90 (0.70)	3.88 (0.53)	202.00	0.96	−0.05
Teaching RESCUR concepts—purpose	To what extent did the leaders feel they had the ability to explain the purpose of the lessons	1 = not at all, 5 = completely	1–5	3.96 (0.71)	3.54 (0.78)	145.50	0.12	−1.56
Teaching RESCUR concepts—responsiveness	To what extent did the leaders feel they had the ability to respond to the needs of the children during lessons/activities	1 = not at all, 5 = completely	1–5	3.74 (0.61)	3.58 (0.51)	175.50	0.41	−0.82

### Data Analysis

Descriptive statistics were generated to describe the implementation-related factors, including consideration of fidelity (which was high in both observations and self-reports). The Mann-Whitney *U*-test was used to see if there were significant differences in the implementation quality of RESCUR between the school and the social-services sectors. The significance level (asymptotic) was set at *p* < 0.05. The results are shown in Tables 2, 3.

## Results

### Implementation Outcomes According to the Observation Protocols

Analysis of the observations, which were conducted after ~3 months of implementation, showed that RESCUR was implemented with desirable outcomes in both schools and social services (Table 2). On a scale from 1 to 7, the mean value of the global rating of implementation was ~5.5 in both sectors, which corresponds to a value between “high” and “very high.”

In most cases, i.e., on 7 out of 9 the observation measures, there were no median value differences between the two sectors. However, for two of the implementation aspects, we did find significant median value differences between the sectors. First, school staff scored higher on “Pacing of RESCUR lessons,” i.e., how often the teacher adapted the pace of the RESCUR lesson to ensure that the children could understand, than did social services staff (*p* < 0.01). Second, social services staff scored higher on “Interpersonal style” (i.e., interest and/or affection toward students and sensitivity to the needs of individual students) than did school staff (*p* = 0.02).

### Implementation Outcomes According to Leaders' Self-Reports

In line with what was found from the observation protocols, analysis of RESCUR leaders' self-reports showed that RESCUR was implemented with desirable outcomes in both schools and social services (Table 3 shows mean values for all outcomes). All aspects of implementation outcomes were in the top half of the possible ranges according to their mean values. At the same time, the self-reports' median values differed significantly between school and social services staff on five out of the 14 aspects of implementation. First, social services staff reported having delivered more (*p* = 0.04) and longer (*p* < 0.01) lessons than did school staff (two aspects of dosage). Second, school staff reported higher fidelity to (*p* = 0.02) and less adaptation of (*p* < 0.01) the intervention than did social services staff (two aspects of fidelity). Finally, school staff reported higher child responsiveness in terms of engagement (*p* = 0.04) than did social services staff. There was no difference with regard to any of the teaching items according to the self-reports.

## Discussion

This study aimed to examine the scaling-out of RESCUR and, more specifically, to test whether the implementation outcomes differed between the school and social services sectors. Studies show that implementation is crucial to the outcome of an intervention (32). Durlak (32) points out that it is extremely costly to ignore the issue of implementation, and that the monitoring of implementation is an essential component of all program evaluation. Here, we have mentioned three studies to give examples of different kinds of implementation, two European and one American. It seems as if quality of implementation is just as important for programs with a manual to follow (SET and PATHS) as for those without (SEAL). It also seems like teachers are themselves affected when implementing a social and emotional program (21).

Many health promotion interventions are conducted in schools (30, 33), but we wanted to examine the implementation of RESCUR in Sweden in two settings and investigate whether it is possible to scale-out RESCUR, which was designed to be used in schools, to another delivery system, i.e., social services. RESCUR was developed to meet the vulnerability of children who encounter psychosocial stressors, and it would be beneficial if there were additional arenas in which they can be addressed. Both observation protocols and group leaders' self-reports showed that RESCUR was implemented with desirable outcomes in both schools and the social services. This suggests that RESCUR can be successfully implemented in different delivery systems, and that the social services sector may be one of them.

The study adds to the literature on the scaling-out of school-based health-related interventions (11). To our knowledge, it is unique because the scaling-out of implementation from the school to other sectors has not been reported on previously. Since implementation is an important factor in the success of a program (30), scaling-out is a necessary first step in the process of finding out whether RESCUR can be used to promote resilience in other settings.

In this study, we focused on five central aspects of implementation (7–9): fidelity, adaptation, dosage, quality of delivery, and participant responsiveness. Overall, implementation in all aspects was high in both settings. Implementation was in line with that found in previous studies of resilience, or similar interventions, conducted in the school setting (7, 20). Participant responsiveness was somewhat higher in this study than what has been found in earlier work (20). Previous studies of the implementation of programs designed to be used to promote resilience among students at school in a social services setting are lacking. Therefore, comparisons of implementation in the social services setting could not be compared with findings specifically in that setting.

Generally, implementation did not differ between the two delivery systems (i.e., school and social services). We found only two main differences on the teacher-observation forms: (1) in the leaders' ability to pace RESCUR lessons/activities to ensure children's understanding; and (2) in the leaders' interpersonal style, i.e., their ability to demonstrate warmth, and to give positive feedback to and build relationships with the children. School staff were better at pacing the lessons, whereas social workers scored higher on interpersonal style as leaders. We found four categories of differences regarding implementation in the leaders' self-reports: (1) Fidelity to guidelines—the leaders' ability to follow the teacher manual; (2) Adaptation—whether the leaders made any changes to the program (another aspect of fidelity); (3) Dosage—how many lessons in total and how much time was spent on each lesson (separate items on the self-report forms); and (4) Children's responsiveness and engagement throughout RESCUR. School staff adhered more to the manual and made fewer adaptations than social services staff. Social services staff provided more and longer sessions than school staff. Finally, school staff reported higher engagement among children than did social services staff.

How could it be the case that RESCUR was so well-implemented in the social services sector by their own staff, when it was specifically designed to be implemented in schools by teachers? In previous studies of the factors that influence implementation on program outcomes (2, 34, 35), fidelity, quality and dosage are often reported as the key components. Fidelity, quality and dosage are measured by adherence to the program protocol, the amount of the program delivered, the quality of program delivery, and participants' reaction and acceptance. These were all implemented with desirable outcomes, and are therefore important variables that make implementation successful. Another important aspect that influences implementation is the context and the frameworks within which an implementation is sanctioned (2, 10). This study structured the implementation according to the four “Quality Implementations Framework” phases in close collaboration with the host settings (schools and social services). We had a clear structure for implementation—initially, ongoing, and for future applications. Furthermore, the study had important frameworks that supported implementation, such as financial resources, organizational support, social relations and support from leaders in both the arenas. All in all, the study shows that the key to implementation lies at a multi-dimensional level, and it is important to address all of the above to have desirable implementation outcomes.

Regarding differences in fidelity and dosage between schools and social services, our results showed that leaders in the social services sector were more likely to make changes to the program than school teachers, and that leaders in the social services sector spent more time on each lesson than school teachers. These are interesting findings, since these factors do not seem to have impacted the overall result of the implementation. One possible explanation is that the children in the social services groups were in stressful psychosocial life situations, which meant that leaders could not always follow the manual and had to give them more time during the lessons. By contrast, the school schedule does not often enable teachers to extend any lesson, so this option might not have been available to them, even if they wanted to pursue it. The size of the groups in social services were smaller, which might have made it easier for the leaders to build a personal relationship with the children. On the other hand, the teachers knew the children in their classes, so therefore the relationship was already established. All in all, it seems as if the changes have not impacted overall fidelity but might be relevant to adjustment of the program protocol.

Regarding the pacing of RESCUR lessons, it was found that school teachers adapted program content to ensure children's understanding more often than leaders in the social services. This may have been expected since teachers would have had more training in adapting a program's content to children's learning levels and have had more experience of educational procedures. Whether the deliverers of an intervention should adapt their manner of delivery depends a lot on their confidence and skills. If implementers have a good understanding of an intervention, and if they have enough confidence, adaptation may be preferable to fidelity (4).

What other factors might have impacted the implementation? In the overall trial of RESCUR, two members of the research team, both with several years of working experience within the two organizations, worked as educators and observers. Support for leaders might have been an important factor in enabling scaling-out to a different delivery system. Regarding the finding that leaders in the social services sector demonstrated more warmth toward the children (of roughly the same age in school and social services), this may be explained by the fact that group sizes differed. Groups in social services were a lot smaller than the classes in schools. Another possible explanation is that social work professionals are specifically trained in empathic meetings. Since we wanted to implement RESCUR in the natural setting of a particular delivery system, group sizes were not moderated, but the effects of such contextual moderation could be further analyzed in future implementation research.

### Strengths and Limitations

One strength of this study is that it was performed under ordinary conditions with regular staff trained in the RESCUR program. But this also means that certain limitations need to be considered when interpreting the results. There are several obvious variables that we were not able to control for, and do not know if they had any impact on the results. It became clear that the municipalities in the study had different resources, and that this may have affected the amounts of effort, time and dedication that leaders put into the implementation of RESCUR. In some schools, RESCUR collided in time with other assignments or with sick leave among the personnel who were supposed to be responsible for implementation.

Regarding study design, a weakness was that two of the educators were also the observers, who each conducted their observations in just one of the two sectors. Before the start of the observations, the observers calibrated their observational scoring, but the study could have been improved by having more independent observers. On the other hand, since the results from the leaders' self-reports are similar to those from the observations, it seems as if this part of the study design did not have a large impact or create a bias. Having multiple methods for measuring implementation is a strength of the study.

Furthermore, in the existing literature, most studies report on the *efficacy* of an intervention, i.e., on its performance under controlled conditions with extensive training and supervision. More research needs to be performed on the *effectiveness* of an intervention, meaning its performance under real-world conditions. As already mentioned, the intervention was delivered in a “natural” setting, with voluntary participation and by leaders who still had their ordinary work assignments to keep up with. This approach reduces researcher control over confounding factors, increasing the risk that inadequate implementation leads to poor results of what otherwise would have been an effective intervention. However, the ecological validity of the study is increased, and studies based on this approach are a pre-requisite for justifying broad implementation.

A final limitation concerns the use of single level analyses. In this type of research, multi level analyses provide a strong tool to control for the effects of group membership. However, in our specific case, we concluded that statistical power might have been an issue (36, 37).

### Future Directions

Benefit, cost-efficiency relative to context and compatibility, and the fit of an intervention to specific goal achievement are particularly important in influencing the pace of implementation (31). RESCUR was designed to be used in contexts where all kinds of children are present, and was therefore made cost-effective, observable and understandable to a large number of professionals. The findings of this study indicate that RESCUR can also be used as a more targeted intervention within social services. In the future, it would be interesting to study the program in the social services sector with regard to its cost-efficiency.

Now that implementation quality has been established, there is a need for future research regarding how a RESCUR intervention might influence the resilience and mental health of children, and whether there are any differences in outcomes between schools and social services. Such research is the next step in our research program.

## Data Availability Statement

The dataset generated and analyzed during the current study is not publicly available, but is available from the corresponding author on reasonable request.

## Ethics Statement

The studies involving human participants were reviewed and approved by Study reviewed and approved by the Regional Ethical Review Board in Uppsala (Dnr 2016/460) on March 15, 2017. Study transferred to the Regional Ethical Review Board in Göteborg (Dnr T523-17) on July 5, 2017. The patients/participants provided their written informed consent to participate in this study.

## Author's Note

JLL is a licensed psychologist specializing in clinical psychology and has a PhD in psychology. BK has a PhD in public health, is a special education teacher, and is a licensed psychotherapist and senior consultant at the Department of Clinical Sciences, Umeå University. CE is professor emeritus in public health, now guest researcher at the Department of Public Health Sciences, Stockholm University. BH is a registered occupational therapist, who has worked with the leading, training, and supervision of support groups for children of alcoholics over the last 30 years. TS is a senior lecturer and associate professor at the Department of Psychology, University of Gothenburg, Sweden.

## Author Contributions

BK, CE, and TS planned and designed the research program. JLL and TS conceived the present study and carried out the statistical analyses. JLL drafted the manuscript with support from TS. BK and BH performed the data collection and critically reviewed the manuscript. CE critically reviewed the manuscript. All authors read and approved the final manuscript.

## Conflict of Interest

BK and CE co-authored the RESCUR program. The remaining authors declare that the research was conducted in the absence of any commercial or financial relationships that could be construed as a potential conflict of interest.

## Abbreviations

PATHS: Promoting Alternative Thinking Strategies
RESCUR: Resilience Curriculum
SEAL: Social and Emotional Aspects of Learning
SET: Social and Emotional Training.
